# Identification of a human immunodominant T-cell epitope of *mycobacterium tuberculosis *antigen PPE44

**DOI:** 10.1186/1471-2180-11-167

**Published:** 2011-07-25

**Authors:** Barbara Cuccu, Giulia Freer, Alessandro Genovesi, Carlo Garzelli, Laura Rindi

**Affiliations:** 1 Dipartimento di Patologia Sperimentale, Biotecnologie Mediche, Infettivologia ed Epidemiologia, Università di Pisa, I-56127 Pisa, Italy

## Abstract

**Background:**

Recently our group has identified a novel antigen of *Mycobacterium tuberculosis*, protein PPE44, belonging to the "PPE protein" family. Although its role in infection is largely unknown, PPE44-specific immune responses were detected in mice infected with *M. tuberculosis*; moreover, immunization of mice with PPE44 subunit vaccines resulted in protective efficacy comparable to the one afforded by BCG against *M. tuberculosis *(Romano *et al*., Vaccine 26, 6053-6063, 2008).

**Results:**

In the present paper, we investigated anti-PPE44 T-lymphocyte responses during human infection by evaluating the frequency of PPE44-specific interferon (IFN)-γ-secreting cells by ELISpot and flow cytometry in a small cohort of healthy subjects that had proven positive to PPD (PPD^+^) *in vitro*, in patients with active tuberculosis, in subjects vaccinated with BCG and in unvaccinated, PPD^- ^healthy controls. We showed IFN-γ^+ ^T cell immune responses to recombinant PPE44 in at least a very high proportion of PPD^+ ^individuals tested and, to a lower extent, in subjects vaccinated with BCG. By the use of a panel of overlapping synthetic 20-mer peptides spanning the PPE44 primary amino acid sequence, we identified a strong CD4^+ ^T-cell epitope, encompassed by peptide p1L (VDFGALPPEVNSARMYGGAG), in the NH_2_-terminus of the PPE44 molecule at the amino acid position 1-20. Conversely, our experiments did not provide evidence of a significant IFN-γ^+ ^CD4^+ ^T cell response to PPE44 or its immunodominant peptide p1L in most (7 out of 8) patients with active TB.

**Conclusions:**

Our data suggest an important immunological role of PPE44 and its immunodominant epitope p1L that could be useful in the design of anti-tuberculosis vaccines and in the immunological diagnosis of *M. tuberculosis *infection.

## Background

Tuberculosis (TB) is the most significant bacterial infection of humans worldwide involving an estimated 2 billion people, that is one third of the world's population [1]. The host's immune system plays a central role in the progression of TB infection; it is in fact estimated that about 5-10% of individuals that become infected with *Mycobacterium tuberculosis *develop active pulmonary TB and become infectious, while the large majority develop latent infection due to the immunological containment of infection in specific granulomas where tubercle bacilli do not multiply, but persist in a dormant state without provoking any clinical symptoms [2,3]. Latent TB may undergo reactivation when the immune system is less efficient, for example due to HIV infection, malnutrition, aging or other causes. As it is estimated that 1 in 10 individuals infected with *M. tuberculosis *will develop active TB in their lifetime [4], latent infection represents a huge reservoir for new TB cases.

At present, the main strategies pursued to improve TB control are more rapid case-finding, efficient drug treatment and the development of a new TB vaccine, more effective than the currently available *Mycobacterium bovis *bacille Calmette-Guérin (BCG). There is therefore a pressing need to detect new TB antigens to set up sensitive immunological tests that may improve the identification of latent TB and to develop effective vaccines capable of activating the immune responses relevant for protection. A Th1-type immune response, based on MHC class II-restricted *M. tuberculosis*-specific CD4^+ ^T cells producing IFN-γ, is considered essential for immunological containment of *M. tuberculosis *infection, although different immune cell subsets, such as αβ^+ ^CD8^+ ^or γδ^+ ^T cells, or other unconventional T cells, namely CD1-restricted αβ^+ ^T cells, contribute to immune protection [5,6].

In the last years, our group has identified a novel antigen of *M. tuberculosis*, protein PPE44 (Rv2770c), belonging to the "PPE proteins", a family of 69 polymorphic proteins of *M. tuberculosis*, defined on the basis of the amino acid (aa) motif Pro-Pro-Glu. Together with the PE (Pro-Glu) proteins, they account for approximately 10% of the coding capacity of *M. tuberculosis *genome [7]. PPE proteins are characterized by a conserved NH_2_-terminus domain of approximately 180 aa residues and a C-terminal domain variable in sequence and length; although their role in *M. tuberculosis *infection is unknown, their polymorphic nature suggests that they represent antigens of immunological relevance [8]. In our past studies, we reported that infection of mice with BCG or with *M. tuberculosis *induced PPE44-specific humoral and cellular immune responses [9,10] and, most importantly, vaccination of mice with PPE44-based subunit vaccines followed by an intratracheal challenge with virulent *M. tuberculosis *resulted in protective efficacy comparable to that afforded by BCG [10]. This finding makes PPE44 a promising antigen candidate for TB subunit vaccines.

In the present work, we evaluated the cellular immune response to PPE44 during mycobacterial infection by determining the T-cell response to PPE44 in a small cohort of subjects. Moreover, by the use of synthetic peptides spanning the PPE44 molecule, we mapped a human immunodominant epitope potentially useful for the development of new subunit TB vaccines and immunological diagnosis of TB.

## Results

### Human T cell response to rPPE44

**Figure 1:**
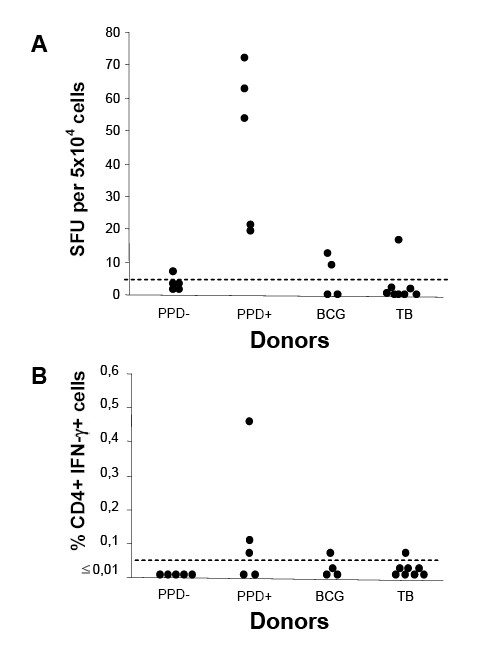
**IFN-γ secretion by PBMC from PPD^-^, PPD^+ ^and BCG-vaccinated healthy donors and from patients with active TB in the presence of rPPE44, as determined by ELISpot (panel A) and ICC (panel B)**. ELISpot results are expressed as spot-forming units (SFU) per 5 × 10^4 ^cells; SFU values above 5, indicated by a horizontal dotted cut-off line, were considered as positive responses. ICC flow cytometry results are expressed as the % of IFN-γ^+ ^CD4^+ ^cells after subtracting background (% of IFN-γ^+ ^CD4^+ ^in the negative controls). Values above an arbitrary cut-off of 0.01% are classified as positive.

We first determined the number of PBMC producing IFN-γ to recombinant PPE44 (rPPE44) by ELISpot in 5 PPD^- ^(negative controls), 5 PPD^+^, 4 BCG-vaccinated subjects and 8 patients with active TB. As shown in Figure 1A, no IFN-γ-secreting spots were observed in any but one PPD^- ^healthy donors; two out of 4 subjects vaccinated with BCG responded to rPPE44 by producing 10 and 16 spots per 5 × 10^4 ^cells, respectively. All healthy PPD^+ ^individuals responded to rPPE44 yielding the highest numbers (18-71) of IFN-γ-secreting spots. Importantly, for patients with active TB, the responders to rPPE44, as well as the numbers of IFN-γ SFU, were significantly lower (P < 0.005, at least) than PPD^+ ^subjects, as only 1 of 8 responded to rPPE44 yielding relatively few spots (13 SFU).

To ascertain that PPE44-specific responses were accounted by CD4^+ ^T cells, we performed ICC assays measuring the frequency of PPE44-specific CD4^+ ^T cells producing IFN-γ. As shown in Figure 1B, the frequency of PPE44-specific CD4^+ ^T cells producing IFN-γ was lower than cut-off in all PPD^- ^healthy donors; 3 out of 5 PPD^+ ^healthy donors yielded the highest positive responses (0.46%). These results probably reflect the lower sensitivity of flow cytometry compared to ELISpot, as shown by other authors as well [11].

### Human T cell responses to PPE44 synthetic peptides

**Figure 2:**
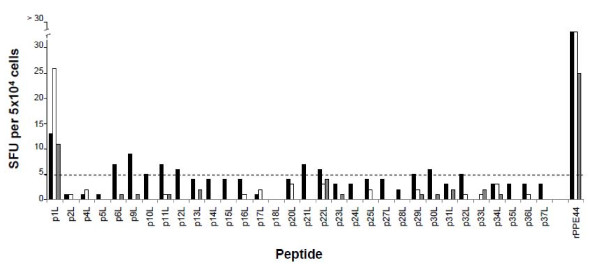
**IFN-γ secretion by PBMC from 3 PPD^+ ^healthy donors in the presence of synthetic 20-mer peptides and rPPE44 (positive control), as determined by ELISpot**. Individual responses to the peptides are indicated as solid, grey and empty bars. Results are expressed as in Fig. 1A.

The next experiments were aimed at mapping PPE44 T-cell epitope(s) by studying T-cell immune response in 3 of 5 PPD^+ ^healthy volunteers used in previous experiment; the 3 subjects chosen tested positive to tuberculin-skin test and Quantiferon TB Gold test. Donors' PBMC were stimulated with a panel of synthetic 20-mer peptides, most of which overlapped by 10 aa, spanning most of the 382 aa sequence of PPE44 and peptide-specific immune responses were then evaluated by ELISpot. As shown in Figure 2, PBMC from all the donors reacted with control rPPE44, as expected, generating numbers of IFN-γ-specific SFU ranging from 25 to 95 per 5 × 10^4 ^cells; only one peptide, *i.e.*, peptide p1L (VDFGALPPEVNSARMYGGAG), spanning aa 1-20 of PPE44, was efficiently recognized by PBMC from all the donors. With regards to the other peptides tested, one donor responded weakly to p6L, p9L, p11L, p12L, p21L, p22L and p30L, yielding 6 to 9 peptide-specific SFU per 5 × 10^4 ^cells, while for the other donors spots were generally lower than 5 per 5 × 10^4 ^cells or absent for all peptides other than p1L. No IFN-γ-positive spots were observed towards p18L (SHITNPAGLAHQAAAVGQAG), spanning aa 171-190, in any of the donors tested. Peptide p18L was therefore chosen as a negative control for subsequent experiments.

These results suggested that p1L represents an immunodominant T-cell epitope of protein PPE44.

### Human T cell responses to p1L peptide

**Figure 3:**
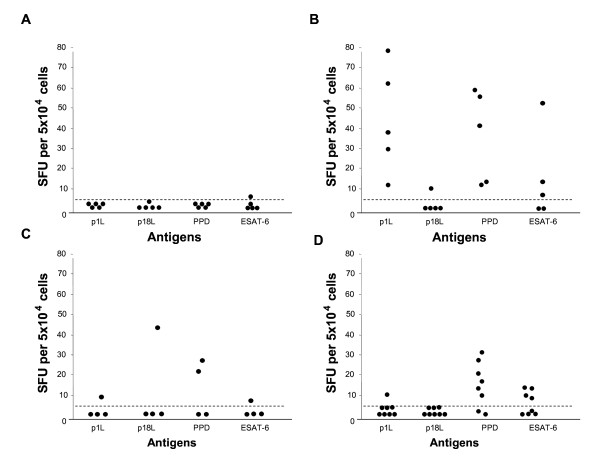
**IFN-γ secretion by PBMC from PPD^- ^(A), PPD^+ ^(B) and BCG-vaccinated (C) healthy donors and from patients with active TB (D) in the presence of p1L, p18L, PPD and ESAT-6, as determined by ELISpot**. Results are expressed as in Fig. 1A.

The T-cell immune response to p1L was then studied in PPD^-^, PPD^+ ^and BCG-vaccinated healthy individuals and in patients with active TB by ELISpot and flow cytometry; PPD and ESAT-6 were included as controls. In PPD^- ^healthy donors, practically no IFN-γ-producing cells were observed in response to p1L, PPD and ESAT-6, as expected (Figure 3A). Conversely, all PPD^+ ^healthy donors (Figure 3B) yielded the highest numbers of IFN-γ-producing cells in response to p1L (13 to 78 spots) and PPD (12 to 58 spots); among the PPD^+ ^healthy donors, 3 out of 5 responded to ESAT-6 (8, 18 and 51 spots, respectively) and one donor responded to control peptide p18L (16 spots) (Figure 3B). A weak IFN-γ response was observed to peptide p1L (11 spots) and antigen ESAT-6 (8 spots) in one of the subjects vaccinated with BCG (Figure 3C); two subjects responded to PPD (22 and 27 spots, respectively) and one subject responded to p18L (45 spots). In the 8 patients with active TB (Figure 3D), the response to p1L peptide was absent or very poor, as only one patient produced a number of IFN-γ-positive spots indicative of an immune response (13 spots). The difference from PPD^+ ^subjects is significant both in terms of proportion of responders and numbers of IFN-γ spots (P < 0.005). Among TB patients, 6 and 4 subjects responded to PPD and ESAT-6, respectively, which is not statistically significant compared to the PPD^+ ^group.

**Figure 4:**
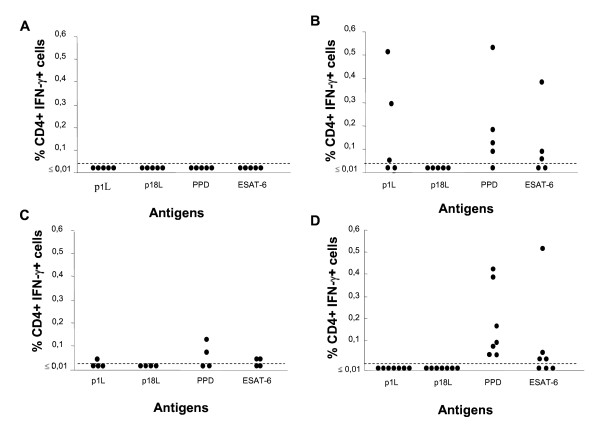
**IFN-γ secretion by PBMC from PPD^- ^(A), PPD^+ ^(B) and BCG-vaccinated (C) healthy donors and from patients with active TB (D) in the presence of p1L, p18L, PPD and ESAT-6, as determined by ICC flow cytometry**. Results are expressed as in Fig. 1B.

On the whole, results obtained by ICC (Figure 4A-D) were comparable to those obtained by ELISpot and confirmed that most PPD^+ ^patients (60% positivity by ICC *versus *100% by ELISpot) had a detectable immune response to p1L peptide, while none of the patients with active TB exhibited a response to p1L peptide. Again, although flow cytometry is less sensitive compared to ELISpot [11], it proves that reacting subjects secrete IFN-γ via their CD4^+ ^T cells. In the responders, the frequency of specific IFN-γ^+ ^T cells was significantly higher than cut-off and reached levels of 0.51%. Among BCG-vaccinated donors, a weak response to p1L was observed in only one donor.

**Figure 5:**
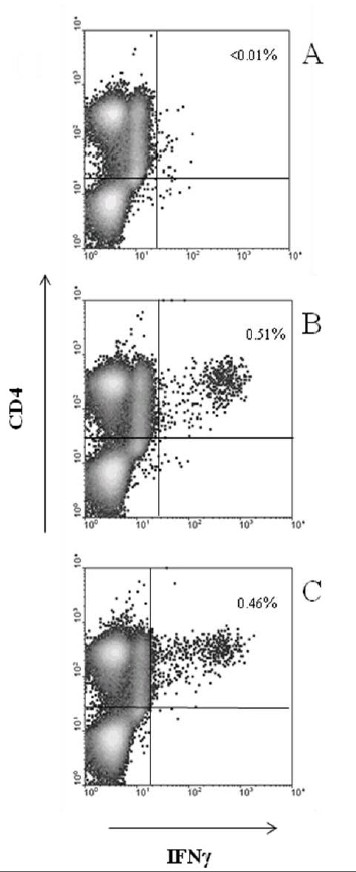
**Representative examples of ICC flow cytometry analysis of PBMC in response to p1L and rPPE44**. The percentage of IFN-γ^+ ^CD4^+ ^cells is given in the upper right corner of each panel. Panel A, PBMC from a PPD^- ^healthy donor in the presence of p1L; panel B and C, PBMC of a PPD^+ ^healthy donor in the presence of p1L and rPPE44, respectively.

An example of the ICC analysis for peptide p1L and rPPE44 of PBMC obtained from a PPD^+ ^donor is given in Figure 5B-C. As can be seen, no reactivity was detected either against p1L, or against rPPE44 in the CD4^- ^population of cells. Thus, p1L is recognized by all PPD^+ ^healthy subjects tested by ELISpot and reactivity is accounted for by CD4^+ ^cells.

## Discussion

The results reported in this paper show that an IFN-γ^+ ^T cell immune response to PPE44 can be detected by ELISpot in all healthy individuals naturally PPD^+ ^and, to a lower extent, in subjects vaccinated with BCG; CD4^+ ^T lymphocytes account for IFN-γ secretion in PPE44-responder subjects, as shown by ICC analysis. By the same approaches, our study has highlighted the presence of a strong CD4^+ ^T-cell epitope in the NH_2_-terminus of the PPE44 molecule localized at the aa position 1-20. Conversely, no significant IFN-γ^+ ^CD4^+ ^T cell response to PPE44 or its immunodominant peptide p1L could be detected in most patients (7 out of 8) with newly diagnosed active TB.

The PPE44 immunodominat T-cell epitope detected in the present study has been previously reported as the antigenic target of an IL-2-induced IFN-γ^+ ^response in mice in which immunization with PPE44-subunit vaccines conferred protective immunity in an experimental model of TB [10]. The data reported in this paper suggest that IFN-γ^+ ^T-cell responses to PPE44 may be associated to immune protection also in human *M. tuberculosis *infection: indeed, IFN-γ^+ ^T-cells specific for the immunodominant PPE44 peptide p1L were detectable in all individuals whose immune system is likely to have determined the containment of infection and prevented progression to active TB disease (PPD^+ ^healthy subjects), as well as in a proportion of BCG-vaccinated subjects. On the other hand, most patients with active TB, *i.e*., those individuals whose immune system failed to contain TB infection, did not respond to PPE44 or p1L. In this respect, however, it has to be considered that TB patients enrolled in our study were under TB chemotherapy, which might have decreased the *M. tuberculosis*-specific IFN-γ responses [12,13]; another explanation might be that PPE44-specific T cells are sequestered at the site of mycobacterial replication, usually the lung. Alternatively, it is tempting to speculate that the poor T-cell immune responsiveness of TB patients to p1L might be related to a dynamic antigen display due to differential expression of PPE44 during active infection; indeed, in a previous paper, we reported great variations in the expression of the gene coding for PPE44 among *M. tuberculosis *isolates and that only about one third of patients with active TB produced antibodies to PPE44 [14]. A last attractive hypothesis could be that a T cell response to p1L/PPE44 helpes individuals to contain TB infection, while those who do not mount such a response are more prone to develop active disease.

One of the promising features of p1L is that it was recognized by all 5 PPD^+ ^healthy individuals tested, as shown by ELISpot, suggesting that p1L is most probably able to bind a number of human HLA-DR alleles. It also proved to be immunodominant in two different species, being a T-cell epitope also in the C57BL/6 strain of mice [10]. "Promiscuous" helper peptides are peptides that can bind a wide range of MHC class II alleles. Within their sequence, they typically have a motif, called P1-P6, where position 1 can be an aromatic or a hydrophobic aa whereas position 6 can be a small or hydrophobic aa [15]. Indeed, such motif can be found in 3 positions in p1L, namely 1-6, 3-8 and 10-6. Promiscuous peptides have been searched for and described both in mycobacterial antigens [16] and in other antigens, such as the malarial circumsporozoite protein [17]. They allow to overcome the problem of the high degree of polymorphism of the HLA-DR molecules expressed in the human population and for such a reason they are ideal candidates for subunit vaccine design and as diagnostic tools. To this aim, future studies will attempt to establish the HLA class II restriction elements binding p1L.

Two other PPE proteins of *M. tuberculosis *have proven capable of inducing protection against *M. tuberculosis *in experimental models, namely i) the PPE14 (Rv0915c/Mtb41), that has shown promising vaccine potential in human clinical trials [18], and ii) the PPE18 (Mtb39A/Rv1196), that is a component of the subunit vaccine Mtb72F. The latter has recently been investigated in clinical trials showing good tolerability and immunogenicity in humans [19,20]. Dillon *et al*. [21] have reported proliferative response towards aa 1-20 of PPE18 in PBMC from PPD-positive human subjects, that is exactly the PPE region were our studies have mapped the CD4^+ ^T-cell epitope. Indeed, the immunodominant p1L domain shows 60 to 85% aa homology with the corresponding sequences of 30 PPE proteins of *M. tuberculosis *and, in particular, p1L shares 14 identical aa with the NH_2_-terminal 20-aa sequence of the protective antigens PPE18 and PPE14. These observations raise the possibility that cross-reactivity might have contributed to the strong immunogenicity of the conserved and homologous NH_2_-terminal regions of the PPE proteins. These considerations make PPE proteins, especially their immunodominant NH_2_-terminal domains, promising antigen candidates for TB subunit vaccine development.

Latent TB infection is conventionally screened for by the more-than-100-year-old tuberculin skin test, that measures *in vivo *reactivity to tuberculin or PPD, a mixture of mycobacterial antigens, some of which common to non-tuberculous mycobacteria and to the vaccine strain *M. bovis *BCG. Recently, assays based on release of IFN-γ by PBMC exposed *in vitro *to *M. tuberculosis*-specific antigens, such as ESAT-6 and CFP-10, have emerged as attractive specific alternatives to tuberculin skin test to distinguish between *M. tuberculosis *infection and BCG vaccination/reactivity to non-tuberculous mycobacteria [22,23]. However, the sensitivity of both tuberculin skin test and IFN-γ-release assays is suboptimal, and none of these tests distinguish between latent infection and active disease [24]. In this context, PPE44 might turn out as a useful reagent for the immunological diagnosis of latent TB and p1L could prove even more useful than the whole recombinant protein becauseT cell reactivity, especially in thawed PBMC, has often been reported to be higher towards synthetic peptides than to recombinant proteins [25]. Our data indicate that a PPE44- or p1L-specific IFN-γ^+ ^T cell-response occurs in naturally PPD^+ ^individuals, who are likely to harbour latent TB infection, and in a proportion of BCG vaccinees tested, but it is not detectable in most of our patients with active TB. These results, although very preliminary, would make p1L a good candidate, in association with the other TB-specific antigens available, to distinguish between latent infection and active disease.

## Conclusions

The present report identifies p1L (PPE44 aa 1-20) as an immunodominant promiscuous peptide that is worth studiyng further both as a vaccine component and as a diagnostic reagent.

## Methods

### Study subjects and ethics statement

Study subjects included 5 purified protein derivative negative (PPD-) and 5 PPD positive (PPD+) healthy donors, 4 subjects vaccinated with *M. bovis *BCG (BCG), and 8 patients with active TB, as shown by culture isolation of *M. tuberculosis*, recruited from Hospital "SS. Giacomo e Cristoforo", Massa, Italy. Reactivity to PPD was determined on PBMC *in vitro *by ELISpot. The study was approved by the Ethics Committee of Hospital "SS. Giacomo e Cristoforo", Massa, Italy and written informed consent was obtained from all subjects.

### Recombinant PPE44, synthetic peptides, and *M. tuberculosis *antigens

rPPE44 was produced in our laboratory; cloning, expression and purification have been previously reported [9].

A panel of 20-mer peptides, overlapping by 10 aa residues, spanning the entire 382-aa PPE44 sequence except for aa 71-80, was synthesized by ProImmune (Oxford, UK); peptide spanning aa 61-80 could not be synthesized due to technical reasons; aa sequence and position of peptides are given in Table 1. Peptides were initially dissolved in DMSO and stock solutions were prepared in RPMI-1640 medium at 1 mg/ml and stored in aliquots at -20°C until use.

**Table 1:** Amino acid sequence of synthetic peptides of PPE44 used in this study

Peptide (position)	Amino acid sequence
p1L (1-20)	VDFGALPPEVNSARMYGGAG
p2L (11-30)	NSARMYGGAGAADLLAAAAA
p4L (31-50)	WNGIAVEVSTAASSVGSVIT
p5L (41-60)	AASSVGSVITRLSTEHWMGP
p6L (51-70)	RLSTEHWMGPASLSMAAAVQ
p9L (81-100)	ESSALAAAQAMASAAAFETA
p10L (91-110)	MASAAAFETAFALTVPPAEV
p11L (101-120)	FALTVPPAEVVANRALLAEL
p12L (111-130)	VANRALLAELTATNILGQNV
p13L (121-140)	TATNILGQNVSAIAATEARY
p14L (131-150)	SAIAATEARYGEMWAQDASA
p15L (141-160)	GEMWAQDASAMYGYAAASAV
p16L (151-170)	MYGYAAASAVAARLNPLTRP
p17L (161-180)	AARLNPLTRPSHITNPAGLA
p18L (171-190)	SHITNPAGLA HQAAAVGQAG
p20L (191-210)	ASAFARQVGLSHLISDVADA
p21L (201-220)	SHLISDVADAVLSFASPVMS
p22L (211-230)	VLSFASPVMSAADTGLEAVR
p23L (221-240)	AADTGLEAVRQFLNLDVPLF
p24L (231-250)	QFLNLDVPLFVESAFHGLGG
p25L (241-260)	VESAFHGLGGVADFATAAIG
p27L (261-280)	NMTLLADAMGTVGGAAPGGG
p28L (271-290)	TVGGAAPGGGAAAAVAHAVA
p29L (281-300)	AAAAVAHAVAPAGVGGTALT
p30L (291-310)	PAGVGGTALTADLGNASVVG
p31L (301-320)	ADLGNASVVGRLSVPASWST
p32L (311-330)	RLSVPASWSTAAPATAAGAA
p33L (321-340)	AAPATAAGAALDGTGWAVPE
p34L (331-350)	LDGTGWAVPEEDGPIAVMPP
p35L (341-360)	EDGPIAVMPPAPGMVVAANS
p36L (351-370)	APGMVVAANSVGADSGPRYG
p37L (363-382)	ADSGPRYGVKPIVMPKHGLF

PPD and ESAT-6 (Early Secretory Antigen T-6) whole recombinant protein were purchased from Statens Serum Institut (Copenhagen, Denmark).

### Peripheral blood mononuclear cells

Peripheral blood mononuclear cells (PBMCs) were isolated from donors' heparinized blood by Lympholyte-H (Cederlane, Canada) density gradient centrifugation. Briefly, blood was layered over an equal volume of Lympholyte-H and centifuged at 800 *g *for 30 minutes at room temperature. The layer of PBMC was removed and washed twice at 500 and 200 *g *for 10 minutes in RPMI-1640 medium. Cells were suspended in culture medium consisting of RPMI-1640 medium supplemented with 2 mM L-glutamine, 100 U/ml penicillin, 100 μg/ml streptomycin and 10% fetal calf serum or 3% autologous plasma. All culture reagents were from Sigma-Aldrich (Milan, Italy)

### Enzyme-linked immunospot assay (ELISpot)

The ELISpot reagent kit was purchased from Pierce Biotechnology (Rockford, USA) and the assay was performed according to the manufacturers' recommendations. In brief, 5 × 10^4 ^PBMC in a 50 μl-volume of culture medium were plated onto multiscreen 96-well polyvinylidene fluoride membrane bottom plates coated with IFN-γ-specific antibody. A 50 μl-volume of medium alone (negative control), medium containing phorbol 12-myristate 13-acetate (PMA)/ionomycin (positive control), or test antigens was added and plates were incubated for 16-24 hr at 37°C in 5% CO_2_. Test antigens were used at the following final concentrations: rPPE44, 10 μg/ml; synthetic peptides, 10 μg/ml; PPD, 10 μg/ml; ESAT-6, 5 μg/ml. PMA and ionomycin were used at 1 ng/ml and 500 ng/ml, respectively. The plates were subsequently washed and incubated for 1 hr with a biotin-labeled anti-human IFN-γ antibody. After a subsequent wash, the plates were incubated with alkaline phosphatase-labeled streptavidin for 1 hr and then developed using a solution of nitro-blue tetrazolium chloride as substrate. Spots were counted using an automated image analysis system ELISpot reader (AID, Strassburg, Germany). Usually, ELISpot results were classified as valid when spots in wells with medium alone were less than 5 and spots in the presence of PMA/ionomycin were greater than 20. T-cell responses to tested antigens were classified as positive when the numbers of spots were greater than 5.

### Intracellular cytokine cytometry

Two × 10^6 ^PBMC were incubated in polypropylene tubes in 0.5 ml of culture medium alone (negative control) or in the same volume of medium containing PMA/ionomycin at final concentrations of 10 ng/ml and 250 ng/ml, respectively (positive control), or test antigens at the following final concentrations: rPPE44, 1 μg/ml; synthetic peptides, 1 μg/ml; PPD, 10 μg/ml; ESAT-6, 5 μg/ml. Costimulatory antibodies CD28 and CD49d (eBioscience, San Diego, CA, USA) at the concentration of 0.5 μg/ml were added to all tubes, except for the PMA/ionomycin tube [26]. After 1-hr activation at 37°C in 5% CO_2_, brefeldin A, 10 μg/ml, (Sigma-Aldrich) was added to each tube. After a 6-hr incubation, cells were fixed in ice with 1 ml of 1% paraformaldehyde in PBS, washed in FACS buffer (PBS, 2% FCS, 0,1% NaN_3_) and permeabilized in 0,1% saponin. Surface and intracellular staining were carried out in the dark for 1 hr with 4 μl PE-labeled anti-CD4 (Miltenyi Biotec, Bergish Gladbach, Germany) and 0.5 μl FITC-labeled anti-IFN-γ (eBioscience) monoclonal antibodies. Cells were finally washed in FACS buffer/0.1% saponin, resuspended in FACS buffer and analyzed by flow cytometry (FACSCan, Becton Dickinson, San Jose, USA). Viable lymphocytes were gated by forward and side light scatter and 250,000 CD4^+ ^lymphocytes events were acquired for each sample and analyzed with the CellQuest software. The frequencies of CD4^+ ^IFN-γ^+ ^events are given as percentages of total CD4^+ ^cells after subtracting background (% CD4^+ ^IFN-γ^+ ^cells in the negative controls). Values above an arbitrary cut-off of 0.01% CD4^+ ^T cells were classified as positive responses on the basis of previous studies of CD4^+ ^T-cell responses to *M. tuberculosis *antigens [25,27].

### Statistical analysis

Fisher exact test was used to compare the numbers of responders and nonresponders to antigenic stimuli; one-way analysis of variance with post tests was used to determine variations among responses. All test were performed by the InStat software package (GraphPad, San Diego, CA, USA); P values less than 0.05 were considered to indicate statistical significance.

## Competing interests

The authors declare that they have no competing interests.

## Authors' contributions

BC and AG performed the experiments. GF partecipated in the study design and revised the manuscript. CG partecipated in the general supervision of the research and critical revision of the manuscript. LR conceived the study, partecipated in its design and drafting and revision of the manuscript. All authors read and approved the final version of the manuscript.
